# Identification of DBC1 as a transcriptional repressor for BRCA1

**DOI:** 10.1038/sj.bjc.6605577

**Published:** 2010-02-16

**Authors:** H Hiraike, O Wada-Hiraike, S Nakagawa, S Koyama, Y Miyamoto, K Sone, M Tanikawa, T Tsuruga, K Nagasaka, Y Matsumoto, K Oda, K Shoji, H Fukuhara, S Saji, K Nakagawa, S Kato, T Yano, Y Taketani

**Affiliations:** 1 Department of Obstetrics and Gynecology, Graduate School of Medicine, The University of Tokyo, Hongo 7-3-1 Bunkyo-ku, Tokyo 113-8655, Japan; 2 Department of Urology, Graduate School of Medicine, The University of Tokyo, Hongo 7-3-1 Bunkyo-ku, Tokyo 113-8655, Japan; 3 Tokyo Metropolitan Cancer and Infectious diseases Center Komagome Hospital, 3-18-22, Honkomagome, Bunkyo-ku, Tokyo 113 8677, Japan; 4 Department of Radiology, Graduate School of Medicine, The University of Tokyo, Hongo 7-3-1 Bunkyo-ku, Tokyo 113-8655, Japan; 5 SORST, Japan Science and Technology, Honcho 4-1-8, Kawaguchi, Saitama 332-0012, Japan; 6 Institute of Molecular and Cellular Biosciences, The University of Tokyo, Yayoi 1-1-1 Bunkyo-ku, Tokyo 113-0034, Japan

**Keywords:** DBC1, BRCA1, interaction, repression

## Abstract

**Background:**

DBC1/KIAA1967 (deleted in breast cancer 1) is a putative tumour-suppressor gene cloned from a heterozygously deleted region in breast cancer specimens. Caspase-dependent processing of DBC1 promotes apoptosis, and depletion of endogenous DBC1 negatively regulates p53-dependent apoptosis through its specific inhibition of SIRT1. Hereditary breast and ovarian cancer susceptibility gene product BRCA1, by binding to the promoter region of SIRT1, is a positive regulator of SIRT1 expression.

**Methods:**

A physical interaction between DBC1 and BRCA1 was investigated both *in vivo* and *in vitro*. To determine the pathophysiological significance of DBC1, its role as a transcriptional factor was studied.

**Results:**

We found a physical interaction between the amino terminus of DBC1 and the carboxyl terminus of BRCA1, also known as the BRCT domain. Endogenous DBC1 and BRCA1 form a complex in the nucleus of intact cells, which is exported to the cytoplasm during ultraviolet-induced apoptosis. We also showed that the expression of DBC1 represses the transcriptional activation function of BRCT by a transient expression assay. The expression of DBC1 also inhibits the transactivation of the SIRT1 promoter mediated by full-length BRCA1.

**Conclusion:**

These results revealed that DBC1 may modulate the cellular functions of BRCA1 and have important implications in the understanding of carcinogenesis in breast tissue.

The gene encoding DBC1 (deleted in breast cancer 1) was identified during a representative differential analysis to search for candidate breast tumour-suppressor genes on a human chromosome 8p21 region that is frequently deleted in breast cancers (Hamaguchi *et al*, 2002). In this study, the expression of DBC2 (deleted in breast cancer 2) was substantially decreased in breast and lung cancer specimens. On the other hand, the expression of DBC1 was not substantially abrogated in cancers from any source. Molecular and cellular functions of DBC1 are currently extensively investigated to reveal the physiological role of DBC1 (Sundararajan *et al*, 2005; Kim *et al*, 2008; Zhao *et al*, 2008; Cha *et al*, 2009). Endogenous DBC1 is a nuclear protein and is thought to localise in the nucleus depending on its nuclear localisation signal (NLS) at the amino terminus. During tumor necrosis factor-*α-*induced apoptosis, DBC1 is translocated to the cytoplasm with loss of the NLS by caspase-dependent cleavage and this cleavage promotes apoptosis because of the death-promoting activity of its carboxyl-terminal coiled-coil domain (Sundararajan *et al*, 2005). Therefore, caspase-dependent cleavage of DBC1 may function as a positive feedback mechanism to promote apoptosis and this would explain how DBC1 functions as a tumour suppressor. A recent study demonstrated that DBC1 promotes p53-mediated apoptosis through specific inhibition of SIRT1, the mammalian homologue of yeast silent information regulator 2 (Sir2) (Kim *et al*, 2008; Zhao *et al*, 2008). However, functions of DBC1 in living cells still remain largely unknown and it should be determined whether DBC1 has a pivotal role in tumour suppression.

It is well known that the germ-line mutation of BRCA1 predisposes women to early-onset breast and ovarian cancer. BRCA1 is predominantly located in the nucleus and is involved in the basal transcriptional machinery (Scully *et al*, 1997; Anderson *et al*, 1998). BRCA1 regulates stress-inducible gene expressions such as p21 (Ouchi *et al*, 1998), p53 (Somasundaram *et al*, 1999), and GADD45 (Jin *et al*, 2000). The carboxyl-terminal BRCA1, referred to as the BRCT domain, has been shown to be involved in double-stranded DNA repair and homologous recombination (Callebaut and Mornon, 1997; Moynahan *et al*, 1999; Zhong *et al*, 1999). BRCT is indispensable for normal cellular growth because the targeted deletion of the BRCT domain results in embryonic lethality (Hohenstein *et al*, 2001). The major function of BRCT is thought to be a gene regulator, mediating BRCA1 function as a tumour suppressor. This hypothesis is based on several lines of evidence, including that the autonomous transactivation function of BRCT was preserved in a recombinant protein consisting of the BRCT domain fused to a GAL4 DNA binding domain (Miyake *et al*, 2000). In addition, point mutations in the BRCT domain derived from patients with inherited breast cancer result in loss of transcriptional activity, and BRCA1 can also function as a negative regulator on some gene promoters (Chapman and Verma, 1996; Monteiro *et al*, 1996). This domain has already been shown to be an interaction surface with a number of transcription factors and co-regulators (Saka *et al*, 1997; Yarden and Brody, 1999; Wada *et al*, 2004; Oishi *et al*, 2006). A recent study revealed the interplay between SIRT1 and BRCA1 (Wang *et al*, 2008). BRCA1 was shown to stimulate the expression level of SIRT1 through binding to the specific promoter region of SIRT1, and this interplay prompted us to search for the cross talk between DBC1 and BRCA1.

To better understand the functional significance and the transcriptional regulation of BRCA1, we investigated the physical interaction between BRCA1 and DBC1. We found that DBC1 directly interacted with the BRCT domain. Our findings revealed that the amino terminus of DBC1 binds directly to the BRCT domain both *in vitro* and *in vivo*. We studied the effect of the transcriptional regulation of BRCA1 driven by DBC1. These findings establish a principal biological function of DBC1 in the modulation of BRCA1 function, and further identify DBC1 as a possible determinant and potential therapeutic target in breast cancer.

## Materials and methods

### Cell culture

Human cervical adenocarcinoma HeLa (CCl-2), human breast cancer MCF-7 (HTB-22), and human kidney 293T (CRL-11268) cell lines were purchased from the American Type Culture Collection (Manassas, VA, USA). These cells were maintained in Dulbecco's modified Eagle's medium supplemented with 10% foetal bovine serum.

### Plasmid construction

BRCA1 expression vectors, BRCT vectors, and reporter constructs (17M8-AdMLP-luc) were described previously by Wada *et al*, 2004. DBC1 (Clone ID 5496068) and SIRT1 (Clone ID 4518906) expression vectors were purchased from Thermo Fisher Scientific Open Biosystems (Huntsville, AL, USA). Fragments of DBC1 were inserted into pcDNA-Myc vector derived from pcDNA3 (Invitrogen, Carlsbad, CA, USA).

### Chemicals and antibodies

Rabbit polyclonal antibodies were anti-DBC1 (produced in our laboratory) and anti-acetyl-p53 (Upstate, Temecula, CA, USA, catalogue no. 06-758). Mouse monoclonal antibodies were anti-BRCA1 (Calbiochem, EMD Biosciences, Inc., LaJolla, CA, USA, catalogue no. OP93T), anti-Myc (Invitrogen, catalogue no. R95025), and anti-SIRT1 (Abnova, Taipei, Taiwan, catalogue no. H00023411-M01). Anti-BRCA1 (catalogue no. sc-642), anti-p21 (catalogue no. sc-397), anti-p53 (catalogue no. sc-126), and anti-actin (catalogue no. sc-47778) were purchased from Santa Cruz Biotechnology, Inc. (Santa Cruz, CA, USA). Alexa Fluor 488-conjugated donkey anti-mouse IgG (A-21202) and Alexa Fluor 555-conjugated goat anti-rabbit IgG (A-21428) were purchased from Invitrogen.

### Immunoprecipitation and western blot

The formation of a DBC1–BRCA1 complex in HeLa and 293T cells was analysed by immunoprecipitation. The whole-cell extracts of HeLa cells were immunoprecipitated with anti-BRCA1 antibodies, and subsequently immunoblotted by anti-DBC1 antibodies. Reciprocal immunoprecipitation was also performed. Cells (293T) transfected with indicated plasmids were lysed and subjected to anti-FLAG M2 agarose (Sigma Aldrich, St Louis, MO, USA). Immunoprecipitated materials were blotted with anti-Myc antibodies to identify DBC1-containing complexes.

### RNAi

The ablation of DBC1 and BRCA1 was performed by transfection of HeLa cells with small interfering RNA (siRNA) duplex oligos synthesised by Qiagen (Hilden, Germany). Control siRNA (AllStars Negative Control siRNA, Qiagen, 1027281), DBC1-specific siRNA (DBC1-RNAi: 5′-AAACGGAGCCUACUGAACA-3′, which covered mRNA regions of nucleotides 1379–1397 (amino acids 460–466) of DBC1, and KIAA1967-RNAi, SI00461853), and BRCA1-specific siRNA (#14 (SI02664361) and #15 (SI02664368)) were transfected using HyperFect reagent (Qiagen).

### GST pull-down assay

Glutathione *S*-transferase (GST) fusion proteins or GST alone were expressed in *Escherichia coli* and immobilised on glutathione–sepharose 4B beads (GE Healthcare UK Ltd., Buckinghamshire, UK). GST proteins were incubated with [^35^S] methionine-labelled proteins using a TNT-coupled transcription–translation system (Promega Co., Madison, WI, USA). Unbound proteins were removed and specifically bound proteins were eluted and analysed by SDS polyacrylamide gel electrophoresis.

### Luciferase assay and mammalian two-hybrid assay

Transfection was performed with Effectene reagent (Qiagen) according to the manufacturer's recommendation. For luciferase assay, indicated expression vectors and GAL4 vectors were co-transfected with 17M8-AdMLP-luc or SIRT1-luc. For mammalian two-hybrid assay, GAL4 vectors and VP16 vectors were co-transfected. As an internal control to equalise transfection efficiency, phRL CMV-*Renilla* vector (Promega Co.) was also transfected in all experiments. Individual transfections, each consisting of triplicate wells, were repeated at least three times (Wada *et al*, 2004).

### Fluorescence microscopy

Cells (MCF-7) were grown on 12 mm BD BioCoat glass coverslips (BD Biosciences, NJ, USA, 354085) in six-well plates before induction of apoptosis. The cells were treated or not treated with irradiation of ultraviolet (UV) light (0.24 J), fixed with phosphate-buffered saline (PBS) containing 4% paraformaldehyde, and permeabilised in PBS with 0.2% (v/v) Triton X-100. After blocking, the cells were incubated sequentially with anti-BRCA1 and anti-DBC1 antibodies. Secondary antibodies were Alexa Fluor 488-conjugated donkey anti-mouse IgG and Alexa Fluor 555-conjugated goat anti-rabbit IgG. The slides were briefly counterstained and analysed under a confocal fluorescence microscope (Carl-Zeiss MicroImaging Inc., Oberkochen, Germany). Colocalisation was quantified using LSM7 series-ZEN200x software (Carl-Zeiss MicroImaging Inc.), and the ratio of colocalisation pixels *vs* total pixels in the target area was determined. The degree of colocalisation signal is expressed as mean±standard deviation.

### Immunohistochemistry

The procedure for immunohistochemical study has been described by Wada-Hiraike *et al*, 2006. The primary antibody used was anti-DBC1, and the ChemMate EnVision Detection system (DAKO, Carpinteria, CA, USA) was used to visualise the signal.

### Chromatin immunoprecipitation assay

Soluble HeLa chromatin for PCR amplification was essentially prepared as described by Oishi *et al*, 2006. Subconfluent HeLa cells were crosslinked with 1.5% formaldehyde at room temperature for 15 min, and washed twice with ice-cold PBS. The cell pellet was then resuspended in 0.2 ml lysis buffer and sonicated by Bioruptor UCD-250 (Cosmo Bio, Co., Ltd., Tokyo, Japan). The sheared soluble chromatin was then subjected to immunoprecipitation with specific antibodies and protein G-sepharose with salmon sperm DNA (Upstate). After an extensive wash, the beads were eluted. The eluate was incubated for 6 h at 65°C to reverse the formaldehyde crosslink. The extracted DNA was purified using the QIAquick PCR purification kit (Qiagen). PCR was performed using specific primers (Wang *et al*, 2008).

## Results

### DBC1 and BRCA1 interact *in vivo* and *in vitro*

**Figure 1:**
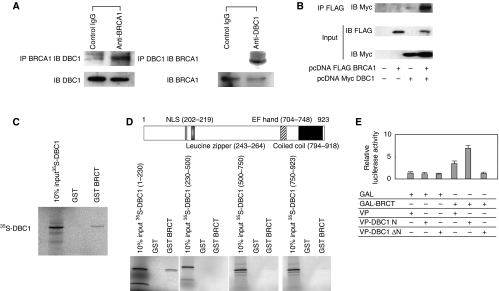
*In vivo* and *in vitro* association between DBC1 and BRCA1, and mapping of the BRCT-interacting region of DBC1. (**A**) The formation of a DBC1–BRCA1 complex in HeLa cells was analysed by co-immunoprecipitation (IP) with antibodies to BRCA1 or preimmune IgG, followed by immunoblotting (IB) using anti-DBC1 antibodies. The immunoprecipitates were subjected to 30 *μ*l of protein G sepharose 4 Fast Flow and bound proteins were detected by western blotting. Reciprocal co-immunoprecipitation with antibodies to DBC1 and subsequent IB confirmed the complex formation of DBC1 and BRCA1. (**B**) The formation of a DBC1–BRCA1 complex in 293T cells was analysed by IP with anti-Flag M2 agarose beads, followed by IB using anti-Myc antibodies. Bound proteins were detected by western blotting. (**C**) Mapping of the BRCT-interacting region of DBC1 using glutathione *S*-transferase (GST)–BRCT and DBC1. Bacterially expressed GST fusion proteins immobilised on beads were used in *in vitro* pull-down assays. Full-length DBC1 was *in vitro* translated in the presence of [^35^S] methionine using a TNT-coupled *in vitro* translation system. Labelled DBC1 was then incubated with GST–BRCT. The mixtures were washed and subjected to SDS polyacrylamide gel electrophoresis (PAGE) and analysed. Polyacrylamide gels were stained briefly with Coomassie Brilliant Blue to verify the loading amounts of fusion proteins. (**D**) A schematic diagram of the structure of DBC1 is shown. Fragments of DBC1 ((amino acids 1–230), (230–500), (500–750), and (750–923)) were *in vitro* translated using a TNT-coupled *in vitro* translation system. Labelled DBC1 was incubated with GST–BRCT. The mixtures were extensively washed and subjected to SDS–PAGE and then analysed by autoradiography. (**E**) Mammalian two-hybrid interaction analysis. Cells (293T) were transfected with the indicated combinations of mammalian expression vectors encoding GAL4, GAL4–BRCT, the herpes simplex virus VP16 transactivation domain (VP16), and VP16-DBC1 chimera. At 24 h after transfection, cells were harvested, and transfected whole-cell lysates were assayed for luciferase activity produced from a co-transfected GAL4 DNA binding site-driven reporter template (17M8-AdMLP-luc). GAL–BRCT shows additive transactivation when co-transfected with VP-DBC1 N, suggesting the interaction between BRCT and DBC1 (1–230) *in vivo*.

To determine the interaction between endogenous DBC1 and BRCA1 in cultured human cells, cell extracts from HeLa cells were immunoprecipitated with anti-BRCA1 antibodies or with preimmune IgG. The immunoblotting analysis using anti-DBC1 antibodies revealed the existence of DBC1 in cell lysate immunoprecipitates (Figure 1A), which indicates that DBC1 physically associates with BRCA1 in living cells. Reciprocal immunoprecipitation analysis confirmed this association (Figure 1A). In addition, Flag-tagged BRCA1 and Myc-tagged DBC1 were each transfected in 293T cells and extracts of transfected cells were immunoprecipitated with anti-FLAG M2 agarose beads. Western blotting analysis with anti-Myc antibodies revealed the existence of Myc-tagged DBC1 in the protein extract of immunoprecipitates (Figure 1B), confirming that BRCA1 was able to form a complex with DBC1. To address the functional importance of the DBC1–BRCA1 interaction, *in vitro-*translated DBC1 in the presence of [^35^S] methionine was incubated with GST and GST fusion BRCT. As clearly shown in Figure 1C, [^35^S]-labelled DBC1 bound the GST-fused BRCT protein, consistent with the results from the immunoprecipitation assay. These data indicated that DBC1 directly interacted with the BRCT domain. To map the region of DBC1 that interacts with the BRCT domain, GST pull-down assays were performed to test for the interaction with GST–BRCT and fragments of *in vitro-*translated DBC1 (Figure 1D). The amino-terminal region of DBC1 including the NLS interacted with the BRCT domain. These findings indicate that the DBC1 amino terminus including the NLS and the BRCT domain are both necessary and sufficient for the interaction between DBC1 and BRCA1. We further confirmed the binding of the BRCT domain to the DBC1 amino terminus using mammalian two-hybrid assays. In this assay, the VP16-fused amino terminus of DBC1 containing the NLS (VP-DBC1 N) exhibited a prominent interaction with the BRCT domain, whereas DBC1 lacking the amino terminus (VP-DBC1 ΔN) showed no interaction, underscoring the results of immunoprecipitation and GST pull-down experiments (Figure 1E).

### DBC1 and BRCA1 colocalise in intact and apoptotic cells

**Figure 2:**
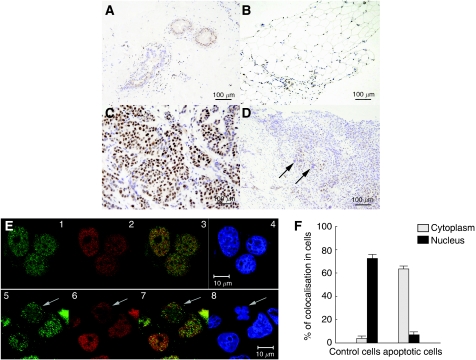
Immunohistochemical detection of DBC1 in human breast tissues and colocalisation of BRCA1 and DBC1 in MCF-7 human breast cancer cells. (**A**–**D**) Breast specimens were obtained at the time of diagnosis of breast cancer in accordance with the guidelines of the Ethical Board of Komagome Hospital. DBC1 showed nuclear staining of ductal epithelium (**A**) and adipose tissue (**B**) in breast specimens. DBC1 expression was observed in the nuclei of cancer tissues (**C**). Cancer cells exhibiting an enlarged nucleus showed a complete loss of DBC1 expression (**D**, arrow). (**E**) MCF-7 cells were either treated or not treated by ultraviolet (UV) light (0.24 J), fixed, and permeabilised. Cells were incubated with primary antibodies and subsequently with secondary antibodies. The expression of DBC1 (red) and BRCA1 (green) was investigated under confocal fluorescence microscopy (Carl-Zeiss). Representative immunofluorescence studies are shown (E, 1–4; control, 5–8; UV exposure for 10 min, E3 and E7; merge, E4 and E8; 4′,6-diamino-2-phenylindole staining). Arrows in E5–8 indicate a cell showing apoptotic morphological changes with the cytoplasmic expression of DBC1 and BRCA1. Bars indicate 10 *μ*m. (**F**) The degree of colocalisation (BRCA1 and DBC1) was measured using a confocal microscope. The colocalisation signal was quantified in the nucleus and cytoplasm of cells separately.

Immunohistochemical studies using human breast specimens showed nuclear staining of DBC1 in the duct and adipose tissue (Figures 2A and B, respectively). Most breast cancer cells exhibited nuclear staining (Figure 2C), but this nuclear staining of DBC1 was not observed in cancer cells with an enlarged nucleus (Figure 2D, arrows). As previously shown, during tumor necrosis factor-*α*-induced apoptosis, DBC1 is translocated to the cytoplasm with a loss of the amino terminus containing the NLS (Sundararajan *et al*, 2005), and the expression of BRCA1 is downregulated by caspase-3-mediated cleavage during UV-induced apoptosis (Zhan *et al*, 2002). The changes in cellular distribution of DBC1 and BRCA1 during apoptotic processes were examined comprehensively under a confocal microscope in MCF-7 cells treated by UV-mediated death signalling. Both DBC1 (Alexa Fluor 555-conjugated anti-rabbit IgG, red) and BRCA1 (Alexa Fluor 488-conjugated anti-mouse IgG, green) were abundantly expressed and colocalised in the nuclei of control cells (Figure 2E, 1–4). In contrast to healthy cells, both DBC1 and BRCA1 are translocated to the cytoplasm in cells showing apoptotic morphological changes (Figure 2E, 5–8). The degree of colocalisation signal was quantified in both control and apoptotic cells, and these data indicated that UV-mediated apoptosis signalling prompted the translocation of these proteins (Figure 2F).

### DBC1 represses the transcriptional activation function of BRCT

**Figure 3:**
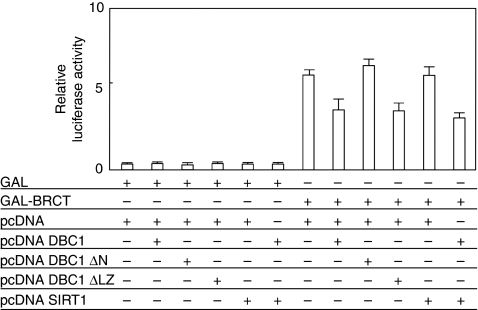
DBC1 represses transcription of GAL4–BRCT through its amino-terminal domain. Transient transfection assays were performed to examine the co-factor activity of DBC1 in the transactivation function of GAL4-fused BRCT. Cells (293T) were transfected with the indicated combinations of mammalian expression plasmids. At 24 h after transfection, the cells were harvested, and transfected whole-cell lysates were assayed for luciferase activity produced from the reporter plasmid (17M8-AdMLP-luc). DBC1 showed a specific repression of the transactivation function of BRCT. The amino terminus of DBC1 was indispensable for this inhibition of BRCT. SIRT1, a binding partner that has roles in cell senescence and tumourigenicity, had no effect on the transactivation function of BRCT. phRL *Renilla* CMV-luc vector was transfected as a control of transfection efficiency. Each experiment was repeated at least three times in triplicate. Error bars represent s.d.

The result that the BRCT domain interacts with DBC1 led us to examine the role of DBC1 in the transactivation function of GAL4-fused BRCT. Transient transfection assays were performed using a 17M8-AdMLP-luc luciferase reporter plasmid, carrying eight tandem repeat GAL4 DNA binding sites (17M × 8) upstream of the major late promoter of adenovirus (AdMLP) driving the expression of the firefly luciferase gene. Although the GAL4–BRCT fusion protein (GAL–BRCT) activated the promoter activity of the reporter plasmid in 293T cells, the transcriptional activity of BRCT was significantly decreased by the expression of DBC1 in luciferase assays (Figure 3). DBC1 lacking an interaction domain with BRCA1 (DBC1 ΔN) lost its ability to inhibit the BRCT-mediated transactivation function (Figure 3). DBC1 lacking a binding region with SIRT1 deacetylase (DBC1 ΔLZ) suppressed the GAL–BRCT transactivation function. SIRT1 showed no influence on the GAL–BRCT transactivation function and on the repression of GAL–BRCT by DBC1. The BRCT-repression function of DBC1 was unaffected in the presence of resveratrol, a major activator of SIRT1, and trichostatin A, a histone deacetylase inhibitor (data not shown). These results suggest that the amino terminus of DBC1 has a significant role in the repression of GAL–BRCT and SIRT1 has no role in regulating the GAL–BRCT function.

### DBC1 disrupts BRCA1-mediated SIRT1 expression

**Figure 4:**
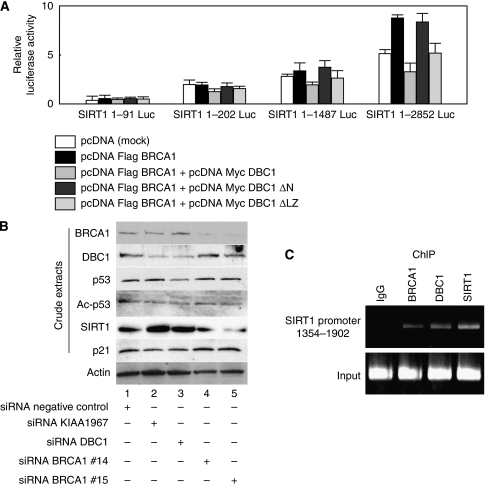
DBC1 represses transcription by BRCA1 through its amino-terminal domain. (**A**) Transient transfection assays were performed to examine the influence of DBC1 using an artificial luciferase reporter construct. Cells (293T) were transfected with the indicated combinations of mammalian expression plasmids. At 24 h after transfection, cells were harvested, and transfected whole-cell lysates were assayed for luciferase activity produced from reporter plasmids. Various lengths of SIRT1 promoter (1–91, 1–202, 1–1487, and 1–2852) were fused upstream of the firefly luciferase reporter plasmid. Full-length DBC1 and DBC1ΔLZ showed specific downregulation of 1–2852 SIRT1-luciferase activity mediated by BRCA1. (**B**) Small interfering RNA (siRNA)-mediated knockdown of BRCA1 decreased the expression of SIRT1. Knockdown of DBC1 resulted in downregulation of acetylated p53 and p21. The expression of SIRT1 was increased by depletion of DBC1. HeLa cells were transfected with indicated siRNA. At 48 h after transfection, cells were harvested and analysed by western blotting. (**C**) Chromatin immunoprecipitation assay was performed to confirm the recruitment of BRCA1 and DBC1 at the SIRT1 gene promoter (1354–1902), a region known to recruit BRCA1 (Wang *et al*, 2008).

The previous chromatin immunoprecipitation assay showed that BRCA1 interacted with the SIRT1 promoter region between 1354 and 1902 and this binding resulted in an elevated expression of SIRT1 (Wang *et al*, 2008). We investigated whether DBC1 has an effect on the BRCA1-mediated stimulation of the SIRT1 promoter. An analysis of the effect of DBC1 on SIRT1-luciferase constructs containing various lengths of SIRT1 promoter regions upstream of the luciferase gene was performed and DBC1 demonstrated a specific downregulation of BRCA1-mediated stimulation on the SIRT1 promoter (SIRT1 1-2852 Luc), evidenced by the expression of DBC1 (Figure 4A). As expected, DBC1 ΔN showed no influence to inhibit the BRCA1-mediated transactivation function of SIRT1-luciferase reporter constructs, whereas DBC1 ΔLZ showed repression on the SIRT1-luciferase transactivation function mediated by BRCA1. We next examined the effect of siRNA-mediated depletion of DBC1 or BRCA1 on their downstream genes. As expected, knockdown of BRCA1 expression by BRCA1-specific siRNA completely abrogated the expression of SIRT1 (Figure 4B, lanes 4 and 5), validating the previous report that the expression of SIRT1 is indeed dependent on BRCA1 (Wang *et al*, 2008). As shown in Figure 4B, lanes 2 and 3, depletion of endogenous DBC1 increased the expression of SIRT1. To demonstrate that SIRT1 functions on the p53 acetylation level (Zhao *et al*, 2008), we tested whether depletion of BRCA1 or DBC1 indeed influences the expression of acetylated p53. Consistent with previous studies (Kim *et al*, 2008; Zhao *et al*, 2008), RNAi-mediated knockdown of DBC1 expression resulted in hypoacetylation of p53 (Figure 4B, lanes 2 and 3), whereas depletion of endogenous BRCA1 had no influence on the expression level of acetylated p53 (Figure 4B, lanes 4 and 5). Depletion of DBC1 resulted in a downregulation of p21, a transcriptional target of BRCA1 (Figure 4B, lanes 2 and 3). Thus, our data demonstrate that DBC1 has a critical role in regulating downstream gene expressions dependent on BRCA1 *in vivo* such as SIRT1 and p21. To test whether DBC1 and BRCA1 were indeed recruited to the SIRT1 promoter, we performed a chromatin immunoprecipitation assay using the SIRT1 gene promoter 1354–1902, a region known to recruit BRCA1 (Wang *et al*, 2008). As expected, a clear recruitment of endogenous BRCA1 to the target sequence (1354–1902) in the SIRT1 promoter was observed in HeLa cells (Figure 4C). Besides this BRCA1 recruitment, DBC1 and SIRT1 were also detected in the promoter region, presumably reflecting the complex formation of BRCA1-DBC1 on the SIRT1 promoter (Figure 4C).

## Discussion

The transcriptional activation function of BRCT is believed to be a key to its tumour-suppressor activity (Chapman and Verma, 1996; Monteiro *et al*, 1996). The importance of BRCT for transcriptional control and growth suppression is also highlighted by the fact that cancer-associated mutations attenuated both, but a neutral polymorphism did not (Humphrey *et al*, 1997; Yarden and Brody, 1999). BRCT possesses an autonomous folding unit defined by conserved clusters of hydrophobic amino acids, and BRCT is likely to represent a protein interaction surface (Saka *et al*, 1997). Although a number of proteins have been identified to interact with the BRCT domain, most of them activate the transcriptional function of BRCT (Wada *et al*, 2004; Oishi *et al*, 2006), and the repressors of BRCT have been poorly studied until now (Chen *et al*, 2001). Here, we clearly showed that endogenous DBC1 associated with BRCA1 *in vivo* and *in vitro*, which suggests the possibility that DBC1 might have a functional relationship with BRCA1-related phenotypical changes. This interaction between BRCA1 and DBC1 was physiologically functional because our results indicated that the DBC1-containing complex might modulate a role of BRCA1 in living cells, repressing BRCT function. In this respect, DBC1 seems to have a tumourigenic role in living cells. The BRCT domain is found in a diverse group of proteins implicated in DNA repair and cell-cycle checkpoint control (Bork *et al*, 1997; Callebaut and Mornon, 1997). A point mutation within the BRCT domain (A1708E) was shown to be critical for DNA damage response by treatment with DNA-damaging agent methylmethane sulphonate (Zhong *et al*, 1999). Thus, repression of BRCT has implications both in tumourigenic and in defective DNA repair processes. Our results also indicated that DBC1 suppressed BRCA1-dependent transcriptional regulation, because SIRT1-luciferase activity was attenuated by the expression of DBC1. Together with the result of chromatin immunoprecipitation assay, these data suggest the possibility that DBC1 might be involved in the basal transcriptional machinery because BRCA1 associates with RNA polymerase II holoenzyme (Scully *et al*, 1997; Anderson *et al*, 1998). DBC1 would serve as a transcriptional repressive factor to manipulate transcriptions, thereby influencing transcriptional products such as SIRT1 and p21. Consistent with these results, our recent data have also shown that DBC1 suppresses the ligand-dependent transcriptional activation function of ER*β* (Koyama *et al*, 2010).

Apoptosis is a normal physiological process that has an important role in embryonic development and in tissue homoeostasis maintenance. As apoptosis is genetically programmed, its dysfunction contributes to tumour promotion. The previous study showed that BRCA1 is cleaved at amino acid 1151–1154 (DLLD) by caspase-3 during UV-C-induced apoptosis, and the cleaved fragment of BRCA1, containing the BRCT domain, induced cell death through activation of BRCA1 downstream effectors, GADD45 and JNK (Jin *et al*, 2000). Another study reported that DBC1 is translocated from the nucleus to the mitochondria during apoptosis (Sundararajan *et al*, 2005). Thus, our results of immunofluoresence showing that the cleavage of DBC1 and BRCA1 after death signalling promotes their cytoplasmic shuttling indicated that BRCA1 and DBC1 may function synergistically in the apoptotic pathway. It seems reasonable to hypothesise that the cells devoid of nuclear DBC1 staining may be apoptotic. Loss of nuclear DBC1 staining in tissues would be speculated as a marker of therapy efficiency.

The accumulation of DNA damage activates p53 and induces cell-cycle arrest and apoptosis. Acetylation of p53 has been shown to augment p53 DNA binding and to regulate the stability of p53 by inhibiting its ubiquitination by MDM2. In response to DNA damage, acetylation of p53 is stimulated and acetylated p53 enhances its ability to induce cell-cycle arrest, apoptosis, and DNA damage repair (Smith and La Thangue, 2005). Consistent with the previous report by Zhao *et al*, 2008, inactivation of endogenous DBC1 leads to hypoacetylation of p53. This would suggest that abrogation of DBC1 causes malfunctions of p53, including defective DNA repair activities. Furthermore, as we discussed above, repression of the BRCT transactivation function may have significance in impaired DNA damage response. The mechanism by which DBC1 regulates DNA damage machinery seems to be complicated, as DBC1 may possess dual roles in promoting and inhibiting DNA repair, because depletion of DBC1 also results in an increased expression of SIRT1, which possesses DNA repair activity (Jeong *et al*, 2007). We have to further confirm the effect of DNA damage response when DBC1 is abrogated. Altogether, our results provide new insight into the fact that DBC1 may serve, at least in part, as a DNA damage response machinery.

In conclusion, our data indicate that DBC1 has an important role in regulating BRCA1-mediated functions through binding to the BRCT domain. In addition to its inhibition of the deacetylase activity of SIRT1, DBC1 represses the expression of SIRT1 by associating with BRCA1. DBC1 may be involved both in tumourigenic and anti-tumourigenic processes. This conflicting mechanism can be the reason why expression of DBC1 was not substantially abrogated in various cancers from any type of tissue (Hamaguchi *et al*, 2002). Therefore, both inhibitors and activators of DBC1 would be therapeutically beneficial, by affecting different DBC1-mediated regulatory pathways together with BRCA1. These results suggest that the failure of binding between BRCA1 and DBC1 may be a key event in cancer predisposition.
